# Surveillance of soil-transmitted helminths and other intestinal parasites in shelter dogs, Mississippi, USA

**DOI:** 10.1016/j.onehlt.2024.100956

**Published:** 2024-12-18

**Authors:** Huan Zhao, Patsy A. Zendejas-Heredia, Vito Colella, Irene Arguello, Kai Brookes, Indu S. Panicker, John M. Williams, Kayla N. Patterson, Gurbaksh Singh, Charlotte V. Hobbs, Richard S. Bradbury

**Affiliations:** a Federation University, Berwick, Melbourne, VIC, Australia; b Department of Veterinary Biosciences, Parkville, Melbourne, VIC, Australia; c Division of Microbiology, University of Mississippi Medical Centre, Jackson, MS, USA; d Department of Paediatrics and Infectious Diseases, University of Mississippi Medical Centre, Jackson, MS, USA; e Division of Paediatric Infectious Diseases, University of Alabama, Birmingham, AL, USA

**Keywords:** Zoonoses, Soil-transmitted helminths, Strongyloidiasis, Toxocariasis, Hookworm infections, Real-time PCR

## Abstract

In recent years, soil-transmitted helminthiases, including strongyloidiasis have become a prominent public health concern in the southeastern United States of America (USA). While there is ongoing human soil-transmitted helminths (STH) surveillance in Mississippi and Alabama, very little attention has been paid to potentially zoonotic STH from dogs in this region. We microscopically examined faecal samples collected from 252 shelter dogs in Mississippi using the formalin-ethyl acetate sedimentation method. Extracted DNA were subjected to three multiplex real-time polymerase chain reaction (qPCR) assays targeting canine STH (canine hookworm species, *Strongyloides* spp.*, Toxocara* species and *Baylisascaris procyonis*). The combined prevalence of STH by microscopy and qPCRs in Mississippi dogs was 62.7 %, with hookworms at 50.0 % and *Toxocara* at 24.2 %. qPCR identified *Ancylostoma caninum* (44.4 %), *Toxocara canis* (22.2 %), *Strongyloides* spp. (1.2 %), and *Uncinaria stenocephala* (0.8 %). No other canine hookworm species, *Baylisascaris procyonis*, or *Toxocara cati* were detected by qPCR. Seven additional intestinal parasites were identified by microscopy, including *Trichuris vulpis* (13.5 %), *Physaloptera* sp. (6.4 %), *Cystoisospora* sp. (3.2 %), *Dipylidium caninum* (1.2 %). *Giardia duodenalis* (0.8 %), *Alaria* sp. (0.4 %), and *Macracanthorhynchus* sp. (0.4 %). These findings, combined with recent human cases in Mississippi, highlight the need for targeted public health messaging to promote regular anthelmintic treatment for dogs and their owners.

## Highlights

• Markedly high prevalence of soil-transmitted helminths in Mississippi shelter dogs.
• *Ancylostoma caninum* is the dominant hookworm species infecting Mississippi shelter dogs.
• *Strongyloides* infection occurs in Mississippi shelter dogs.

## 1 Introduction

Soil-transmitted helminths (STH) represent a major veterinary and human public health concern [1,2]. Several zoonotic STH from canines cause morbidity in humans. *Ancylostoma braziliense* is the causative agent of “creeping eruption” [3]. Eosinophilic enteritis is seen in human infection with *Ancylostoma caninum* [3]. *Ancylostoma ceylanicum* is a notable cause of ancylostomiasis in Southeast Asia, Oceania, and parts of Central America and the Caribbean [[3], [4], [5], [6], [7]]. Rare patent intestinal infections of humans with the dog whipworm, *Trichuris vulpis* have also been reported [8,9]. Toxocariasis, caused by *Toxocara canis* (canine host) and *Toxocara cati* (feline host), manifests as a wide spectrum of disease, including ocular larva migrans (OLM), neural larva migrans (NLM), visceral larva migrans (VLM), and covert toxocariasis, although most infections are asymptomatic [10]. *Strongyloides stercoralis* has been recognised as a potentially zoonotic STH from dogs [11]. Human *S. stercoralis* infection is typically an asymptomatic or paucisymptomatic chronic condition but can develop into a fatal disseminated disease in cases of immunosuppression [12].

In the United States of America (USA), zoonotic STH and strongyloidiasis disproportionately impact marginalised and underserved communities in tropical and subtropical regions, where access to healthcare and adequate sanitation is limited [1,13]. A 2017 study reported molecular evidence of *S. stercoralis* (4/55) and *Necator americanus* (19/55) transmission in a rural county in Alabama, USA [14]. A subsequent survey of 777 children in the same county did not identify cases of STH but revealed a higher *Toxocara* seroprevalence (5 %) than the national level in comparable age ranges [15]. This highlights the possibility of continued focal endemicity of these parasites in the Southeast USA.

In 2019, a cluster of ocular toxocariasis cases was noted from rural Mississippi [16]. Subsequent state-wide serological surveillance revealed increased *Toxocara* seroprevalence among Mississippi residents (9.2 % versus the national average of 5.1 %) [17]. *Strongyloides stercoralis* infections were also identified in this cohort, by serology (4/1960; 0.2 %) and qPCR (1/594; 0.2 %), albeit it is unknown whether these cases were locally acquired [17]. Although unpublished, potentially autochthonous cases of strongyloidiasis in Mississippi have been described (Hobbs, C pers. comm., June 25, 2024). Further adding to the diversity of STH detected in the area, a case of zoonotic ascariasis from Mississippi has recently been reported [18]. Furthermore, despite ongoing cases of creeping eruption in the Southeast USA [19,20], robust epidemiological surveillance for *A. braziliense* in humans and canine reservoirs in this region is scarce [21], with no studies conducted in Mississippi thus far. Collectively, these human infections warrant public health surveillance in domestic dogs (*Canis lupus familiaris*) from Mississippi.

*Baylisascaris procyonis*, an intestinal nematode of raccoons (*Procyonis lotor*), can cause NLM in humans, manifesting as a rapidly fatal eosinophilic meningoencephalitis [22]. Human *B. procyonis* infection is relatively rare in the USA, with only 24 cases documented nationally, including six fatalities [22]. However, the true incidence is likely underestimated due to misdiagnosis or underdiagnosis of cases. When using microscopy for diagnosis, *B. procyonis* eggs can be easily misidentified as *Toxocara* eggs due to their high morphological similarity. A 2016 study of *B. procyonis* seroprevalence in wildlife rehabilitators from the USA found one seropositive case in Mississippi [23]. Rarely, dogs may act as an alternative definitive host of *B. procyonis* [24].

Previous STH surveillance in dogs in the USA have largely relied on faecal flotation [25]. This method is ineffective for detecting *Strongyloides* spp. larvae [26]. When used for hookworm and *Toxocara* detection, microscopy does not allow easy differentiation of species [25]. No study has employed molecular techniques such as real-time PCR (qPCR) for STH surveillance in dogs in the southeastern USA. This may weaken the robustness of epidemiological data on these zoonotic agents in this region. This study aimed to determine the prevalence of STH species in Mississippi shelter dogs using a combination of microscopy and molecular tools. Other canine intestinal parasites of medical and veterinary importance were also included in this investigation.

## 2 Materials and methods

### 2.1 Study sites and sample collection

**Fig. 1:**
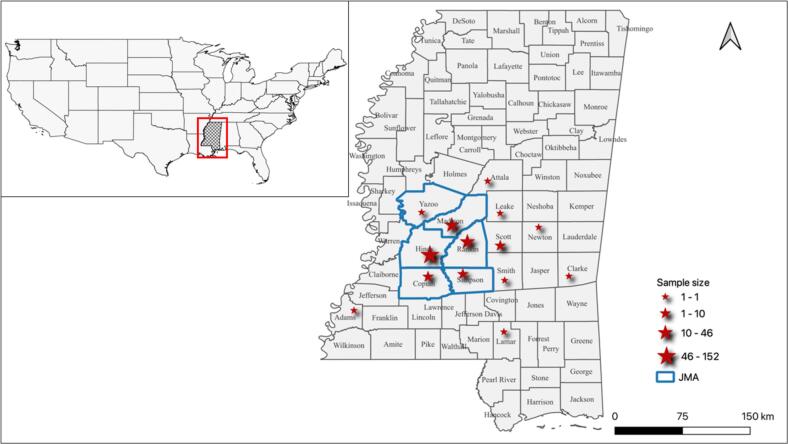
Sampling locations in Mississippi, USA. Red stars indicate sampling locations with their size being proportional to the sample size. The border of counties within the Jackson Metropolitan Area (JMA) is highlighted in blue. (For interpretation of the references to colour in this figure legend, the reader is referred to the web version of this article.)

From June 2019 to January 2020, we collected 252 canine faecal samples from selected animal shelters in 14 counties of Mississippi, USA (Fig. 1). All sampled dogs, including 111 puppies (<1 year of age) and 141 adult dogs (≥1 year of age), had no known history of anthelmintic treatment.

Based on the observed diagnostic prevalence of intestinal parasites (14.4 %) in Mississippi domestic dogs in the same year [33], we estimated that at least 244 samples were required to determine the true parasite prevalence with 95 % confidence level and 5 % precision, assuming a diagnostic sensitivity of 90 % and specificity of 98 %.

Fresh faecal specimens were collected by shelter workers from the ground following dog defecation, and promptly transported to the laboratory at the University of Mississippi Medical Centre. A 250 mg aliquot of faeces was frozen at −80 °C, while the remaining faeces were preserved in sodium acetate-acetic acid-formalin (SAF) (Remel, Lenexa, KS) at a 1:2 specimen-to-fixative ratio and stored at ambient temperature. The preserved faecal samples were subsequently air transported to the Federation University laboratory, Australia for microscopic analysis, and the frozen samples transported to The University of Melbourne, Australia laboratory for molecular analyses.

### 2.2 Laboratory analyses

#### 2.2.1 Microscopic analysis

Faecal parasite concentration was performed on 252 SAF-preserved samples using the formalin-ethyl acetate sedimentation method as previously described [27], with the modification of adding three drops of Triton X-100 (Merck, Darmstadt, Germany) prior to the addition of ethyl acetate. Three entire 22 × 22 mm-coverslip saline preparations of each concentrated faecal deposit were examined by microscopy. Parasite stages were identified based on their morphological characteristics at 100× – 400× magnifications.

#### 2.2.2 DNA extraction

Genomic DNA was extracted from 252 fresh frozen samples (each ≈250 mg) using the QIAmp PowerFaecal Pro DNA Kit (QIAGEN, Hildern, Germany) according to the manufacturer's protocol with minor modifications. Specifically, faecal samples were washed in Milli-Q water and centrifuged at 8000*g* for 60 s followed by decant of supernatant. The bead-beating step consisted of four 30-s cycles with 5-min intervals between each cycle on a FastPrep-24 5G Instrument (MP Biomedicals). DNA was eluted in 100 μL of elution buffer and stored at -20 °C until analysis.

#### 2.2.3 Real-time PCR analysis

We employed two published quadruplex TaqMan qPCR assays to identify *Strongyloides* and canine hookworm species [28,29]. The hookworm-*Strongyloides* qPCR assays were initiated at 95 °C for 2 min, followed by 40 cycles of denaturation at 95 °C for 15 s and annealing at 60 °C for 60 s. All qPCRs were performed in duplicate, incorporating a no-template control and a positive control, using either parasite genomic DNA or gBlocks Gene Fragments. A triplex TaqMan qPCR assay to detect *T. canis, T. cati* and *B. procyonis* was developed (**Supplementary File 1&2**). All primers were as published previously [30]. The published *T. cati* probe [26] was altered by replacement of the Cy5 fluorescent dye with a JOE dye. Additionally, we adapted the previously published *B. procyonis* molecular beacon probe [31] to a ROX labelled TaqMan probe with the fluorescence quencher, 3IAbRQSp. The cycling protocol for the *Toxocara*-*B. procyonis* qPCR included initial activation at 95 °C for 5 min, followed by 40 cycles of denaturation at 95 °C for 10 s, annealing at 60 °C for 15 s and extension at 72 °C for 5 s. Details of the analytical validation of this novel *B. procyonis*-*T. canis*-*T. cati* triplex qPCR are available in supplementary data. Canine mammalian mitochondrial 16S rRNA gene was used as an extraction control and Equine Herpesvirus (EHV)-4 DNA as an amplification control in all qPCR assays.

### 2.3 Statistical analysis

Power analysis was conducted using Epitools (https://epitools.ausvet.com.au/) to determine the minimum sample size required for this survey. Data were analysed using SPSS-29 (https://www.ibm.com/), Microsoft Excel (https://www.microsoft.com/), and GraphPad Prism (https://www.graphpad.com/). Prevalence of STH and other intestinal parasites in faecal samples by microscopy and multiplex qPCR was determined. Cohen's Kappa statistics was used to evaluate the diagnostic agreement between methods for hookworm and *Toxocara* identification, interpreted as “poor” if k ≤ 0.00, “slight” if 0.01 ≤ k ≤ 0.20, “fair” if 0.21 ≤ k ≤ 0.40, “moderate” if 0.41 ≤ k ≤ 0.60, “substantial” if 0.61 ≤ k ≤ 0.80, and “perfect” if 0.81 ≤ k ≤ 1.00 [32]. Chi-square (χ2) analyses were performed to compare parasite prevalence between puppies and adult dogs. A 95 % CI was employed to establish the statistical significance of all results.

### 2.4 Ethical approval

A waiver of approval from the Institutional Animal Use and Care committee at the University of Mississippi Medical Centre was obtained as no direct contact with the animals was undertaken during the collection of these samples. Ethical approval was not required for this study at the Federation University (as per Chair of the Federation University Animal Ethics Committee, February 2020).

## 3 Results

### 3.1 Microscopic surveillance for intestinal parasites

**Fig. 2:**
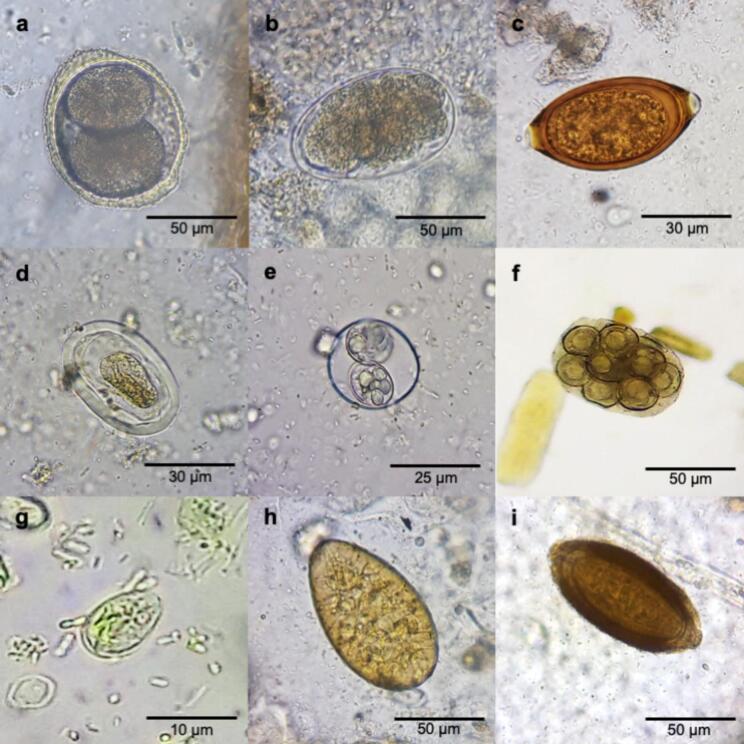
Microscopic images of parasites detected by microscopy at 400× magnification. A) *Toxocara canis* egg; b) hookworm egg; c) *Trichuris vulpis* egg; d) *Physaloptera* sp. egg; e) *Cystoisospora* sp. oocyst; f) *Dipylidium caninum* egg packet; g) *Giardia duodenalis* cyst; h) *Alaria* sp. egg; i) *Macracanthorhynchus* sp. egg.

Microscopic analyses of 252 faecal samples revealed an overall parasite prevalence of 51.6 % (130/252; 95 % CI: 45.2–57.9 %). The most commonly detected parasites were hookworms (31.0 %; 95 % CI: 25.3–37.1 %), *Toxocara* (20.6 %; 95 % CI: 15.8–26.2 %), and *Trichuris vulpis* (13.5 %; 95 % CI: 9.5–18.3 %). Additional identified parasites included *Physaloptera* sp. (6.4 %; 95 % CI: 3.7–10.1 %), *Cystoisospora* sp. (3.2 %; 95 % CI: 1.4–6.2 %), *Dipylidium caninum* (1.2 %; 95 % CI: 0.3–3.4 %), *Giardia duodenalis* (0.8 %; 95 % CI: 0.1–2.8 %), *Alaria* sp. (0.4 %; 95 % CI: 0–2.2 %), and *Macracanthorhynchus* sp. (0.4 %; 95 % CI: 0–2.2 %) (Fig. 2).

### 3.2 Molecular surveillance for soil-transmitted helminths

**Fig. 3:**
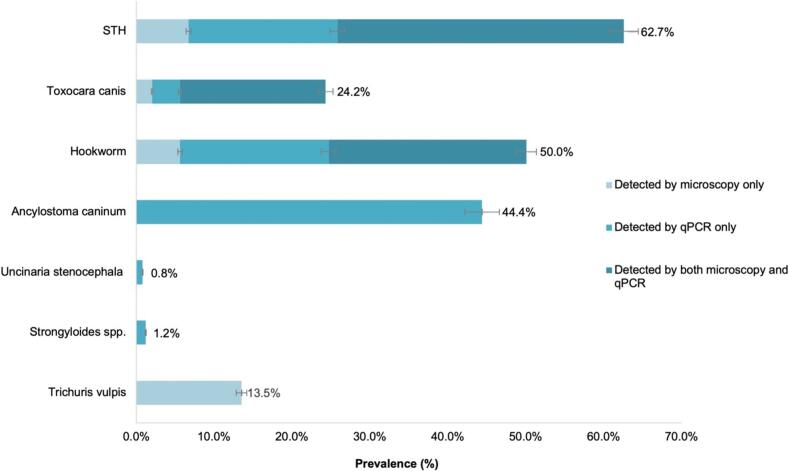
Prevalence of soil-transmitted helminths in Mississippi shelter dogs by microscopy and qPCR (*N* = 252). *Ancylostoma braziliense, Ancylostoma ceylanicum, Toxocara cati*, and *Baylisascaris procyonis* qPCRs were negative. qPCR for the detection of *Trichuris vulpis* was not performed. STH, soil-transmitted helminths.

No samples demonstrated DNA extraction failure or amplification inhibition. Of all 252 samples, 56.0 % (141/252; 95 % CI: 49.6–62.2 %) were positive for at least one zoonotic STH by qPCR. Identified STH were *A. caninum* (44.4 %; 95 % CI: 38.2–50.8 %), *T. canis* (22.2 %; 95 % CI: 17.2–27.9 %), *Strongyloides* spp. (1.2 %; 95 % CI: 0.3–3.4 %) and *U. stenocephala* (0.8 %; 95 % CI: 0.1–2.8 %) (Fig. 3)*.* No *A. braziliense*, *A. ceylanicum*, *B. procyonis* or *T. cati* infections were detected.

### 3.3 Combined microscopic and molecular surveillance for soil-transmitted helminths

**Fig. 4:**
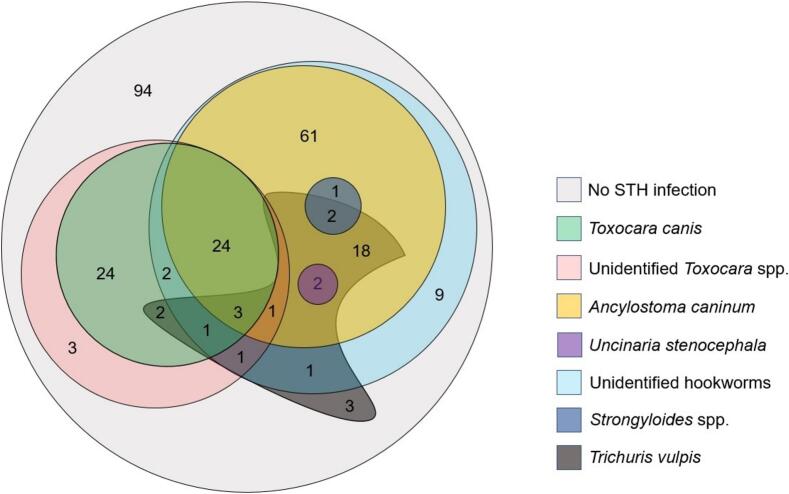
Venn diagram showing the number of canine faecal samples positive for different soil-transmitted helminths (*N* = 252). Overlapping areas indicate STH coinfection. STH, soil-transmitted helminths.

With microscopy and qPCR data combined, an overall 62.7 % (158/252; 95 % CI: 56.4–68.7 %) prevalence of STH in the samples was observed. Infection with single STH was present in 39.7 % (100/252; 95 % CI: 33.6–46.0 %) of the samples, while 23.0 % (58/252; 95 % CI: 18.0–28.7 %) had coinfection (Fig. 4).

*Toxocara canis* was detected in 24.2 % (61/252; 95 % CI: 19.1–30.0 %) of the samples, including 2.0 % (5/252; 95 % CI: 0.7–4.6 %) by microscopy only, 3.6 % (9/252; 95 % CI: 1.7–6.7 %) by qPCR only, and 18.7 % (47/252; 95 % CI: 14.0–24.0 %) by both methods. Hookworms were identified in 50.0 % (126/252; 95 % CI: 43.7–56.3 %) of the samples, including 5.6 % (14/252; 95 % CI: 3.1–9.2 %) by microscopy only, 19.1 % (48/252; 95 % CI:14.4–24.5 %) by qPCR only, and 25.4 % (64/252; 95 % CI: 20.1–31.2 %) by both methods (Fig. 3). Kappa statistics indicated a perfect agreement between qPCR and microscopy for *Toxocara* diagnosis (*k* = 0.84) and a moderate agreement for hookworms (*k* = 0.49).

While *T. canis* was significantly more prevalent in puppies (39.6 %; 44/111; 95 % CI: 30.5–49.4 %) as compared to adult dogs (8.5 %; 12/141; 95 % CI: 4.5–14.4 %) (χ^2^ = 34.820; *p* < 0.01), no statistically significant difference was observed in the prevalence of hookworms or *Strongyloides* spp. between the two age groups. Moreover, *T. vulpis* was significantly less common in puppies (6.3 %; 7/111; 95 % CI: 2.6–12.6 %) than adult dogs (19.2 %; 27/141; 95 % CI: 13.0–26.6 %) (χ^2^ = 8.776; p < 0.01) (Table 1).

**Table 1:** Chi-Square analyses of intestinal parasite combined microscopy and qPCR prevalence in Mississippi shelter dogs by age group (% [95 % CI]).

	Overall prevalence	Age group		
		Puppy (*n* = 111)	Adult dog (*n* = 141)	Chi-Square (χ^2^) statistics
*Toxocara canis*	22.2 [17.2–27.9]	39.6 [30.5–49.4]	8.5 [4.5–14.4]	34.820*
*Toxocara* overall	24.2 [19.1–30.0]	41.4 [32.2–51.2]	10.6 [6.1–16.9]	32.120*
*Ancylostoma caninum*	44.4 [38.2–50.8]	39.6 [30.5–49.6]	48.2 [39.7–56.8]	1.855
*Uncinaria stenocephala*	0.8 [0.1–2.8]	0	1.4 [0.2–5.0]	1.587
Hookworm overall	50.0 [43.7–56.3]	45.1 [35.6–54.8]	53.9 [45.3–62.3]	1.948
*Strongyloides* sp.	1.2 [0.3–3.4]	0	2.1 [0.4–6.1]	2.390
*Trichuris vulpis*	13.5 [9.5–18.3]	6.3 [2.6–12.6]	19.2 [13.0–26.6]	8.776*
*Physaloptera* sp.	6.4 [3.7–10.1]	8.1 [3.8–14.8]	5.0 [2.0–10.0]	1.032
*Cystoisospora* sp.	3.2 [1.4–6.2]	4.5 [1.5–10.2]	2.1 [0.4–6.1]	1.141
*Dipylidium caninum*	1.2 [0.3–3.4]	2.7 [0.6–7.7]	0	3.857
*Giardia duodenalis*	0.8 [0.1–2.8]	1.8 [0.2–6.4]	0	2.561
*Alaria* sp.	0.4 [0–2.2]	0	0.7 [0–3.9]	0.790
*Macracanthorhynchus* sp.	0.4 [0–2.2]	0	0.7 [0–3.9]	0.790
STH	62.7 [56.4–68.7]	64.9 [55.2–73.7]	61.0 [52.4–69.1]	0.398
Single STH infection	39.7 [33.6–46.0]	37.8 [28.8–47.5]	41.1 [32.9–49.7]	0.282
STH coinfection	23.0 [18.0–28.7]	27.0 [19.0–36.3]	19.9 [13.6–27.4]	1.801
Parasites overall	67.1 [60.9–72.8]	72.1 [62.8–80.2]	63.1 [54.6–71.1]	2.253
Single parasitic infection	39.3 [33.2–45.6]	39.6 [30.5–49.4]	39.0 [30.9–47.6]	0.010
Dual parasitic infection	22.6 [17.6–28.3]	27.9 [19.8–37.2]	18.4 [12.4–25.8]	3.194
Triple parasitic infection	4.0 [1.9–7.2]	3.6 [1.0–9.0]	4.3 [1.6–9.0]	0.069
Quadruple parasitic infection	1.2 [0.3–3.4]	0.9 [0–4.9]	1.4 [0.2–5.0]	0.141

Note: *p < 0.01; 95 % CI in square brackets; Abbreviation: STH, Soil-transmitted helminths.

## 4 Discussion

The prevalence of *Toxocara* in Mississippi shelter dogs (24.2 %; 95 % CI: 19.1–30.0 %) substantially exceeded the previously reported US national diagnostic prevalence (1.8–2.0 %) and the Mississippi state prevalence (2.1–5.1 %) in companion dogs [33]. Additionally, a higher prevalence of *S. stercoralis* (1.2 %; 95 % CI: 0.3–3.4 %) was observed in this dog cohort compared to earlier canine surveys across the USA (0.17–0.20 %) [[34], [35], [36]]. Furthermore, hookworm prevalence in Mississippi shelter dogs was considerably higher than reports of CAPC [37] for the same region (5.9 %; 471/7939) and of several microscopy surveys across the southeastern USA (4.0–17.0 %) [[38], [39], [40], [41], [42], [43]], though our microscopy findings (31.0 %; 95 % CI: 25.3–37.1 %) were comparable to a 1996 survey which identified hookworms in 36.5 % (709/1941) of shelter dogs from the southeastern United States [44]. Although *B. procyonis* occasionally infects dogs [24], no cases were identified in this survey, suggesting that shelter dogs in this region may not pose a significant public health risk for baylisascariasis. However, it is important to note that this cohort does not represent all dog populations, and expanded molecular surveillance in a variety of canine hosts is needed to assess the zoonotic risk more comprehensively.

Variations in STH prevalence between studies are partially attributable to differences in diagnostic methods. Prior research has consistently demonstrated the superior sensitivity of qPCR over traditional microscopy for detecting hookworms [28,45] and *Strongyloides* [46]. In our study, combining qPCR and microscopy identified 19.1 % (48/252; 95 % CI:14.4–24.5 %) more hookworm infections, 3.6 % (9/252; 95 % CI: 1.7–6.7 %) more *Toxocara* infections compared to using microscopy alone. All *Strongyloides* infections were detected by qPCR only.

The choice of dog population studied also likely influenced the prevalence. Shelter dogs likely received limited or no veterinary care and anthelmintic treatment prior to the rescue, contributing to higher STH prevalence. Additionally, stray dogs may have additional means of acquiring *Toxocara* or *A. caninum* through greater predation on infected paratenic hosts [33]. Similar patterns were observed in shelter cats from Mississippi, where the prevalence of feline toxocariasis (39 %) and hookworm prevalence (34 %) were 6.5-fold and 11-fold higher, respectively, than the state's diagnostic prevalence [47]. It appears that this high prevalence of canine toxocariasis is spilling over into humans, as indicated by the high seroprevalence of *Toxocara* in Mississippi residents when compared to the United States average [17], and the cluster of *Toxocara* OLM cases recently reported from Mississippi [16].

*Ancylostoma caninum* was the predominant hookworm species infecting Mississippi shelter dogs. Few canine surveys in the USA have identified hookworms to species level [21,39], and one such study by Stafford et al. [39], utilising coproantigen immunoassay, reported a similar result, detecting *A. caninum* in 98.2 % (108/110) of the hookworm-positive samples. *U. stenocephala*, a hookworm that thrives in temperate climate [3], was identified in 0.8 % (2/252) of Mississippi shelter dog samples. Transmission of *U. stenocephala* during cold seasons in the Southeast is possible given that the optimal temperature for free-living development of this parasite was described as 20 °C, above the average winter temperature in Mississippi (15 °C) [48]. No *A. braziliense* were identified, despite its presence in dogs in nearby Florida [21], where it is likely responsible for sporadic cases [49] and outbreaks [50] of creeping eruption in humans. The absence of *A. ceylanicum* suggests that this zoonotic hookworm has not yet reached the South-East of the USA from Central America or the Caribbean. This hypothesis warrants confirmation through broader surveillance of other dog populations and companion animals.

### 4.1 Limitations

This study carries several potential limitations. Firstly, it relies on convenience sampling of shelter dogs, primarily from Hinds (152/252) and Rankin (46/252) counties (Fig. 1), potentially leading to an over-representation of these regions and an under-sampling of others. Secondly, assessing the impact of shelter characteristics on observed parasite prevalence is challenging, as cross-transmission of parasites among co-residing dogs may occur based on housing conditions. This may confound the prevalence observed in this study.

## 5 Conclusion

This sentinel surveillance study of 252 shelter dogs from Mississippi revealed a considerably high prevalence of canine intestinal STH infections. STH identified by microscopy and/or qPCR included *T. canis*, *A. caninum*, *U. stenocephala*, *Strongyloides* spp., and *T. vulpis*. These findings, combined with recent human findings in Mississippi, and emerging reports of anthelmintic drug resistance in *A. caninum* in the USA, indicate a need for enhanced public health messaging regarding regular anthelmintic treatments targeted towards dog owners and the implementation of a targeted One-Health initiatives for STH zoonoses control, particularly in marginalised communities within the state. The use of highly sensitive molecular tools for future canine STH surveillance in Mississippi and the broader American South-East is encouraged.

## Funding

Funding for this study was provided by the University of Mississippi Medical Centre Vice Chancellor's Research funds (CVH).

## CRediT authorship contribution statement

**Huan Zhao:** Writing – original draft, Methodology, Formal analysis, Data curation, Investigation, Validation. **Patsy A. Zendejas-Heredia:** Writing – review & editing, Validation, Methodology, Supervision. **Vito Colella:** Writing – review & editing, Supervision, Methodology, Resources, Validation. **Irene Arguello:** Investigation, Validation. **Kai Brookes:** Investigation, Validation. **Indu S. Panicker:** Writing – review & editing, Supervision, Investigation. **John M. Williams:** Investigation, Supervision, Validation. **Kayla N. Patterson:** Investigation, Supervision. **Gurbaksh Singh:** Investigation, Supervision. **Charlotte V. Hobbs:** Writing – review & editing, Supervision, Resources, Project administration, Funding acquisition, Conceptualization, Methodology. **Richard S. Bradbury:** Writing – review & editing, Supervision, Resources, Project administration, Methodology, Conceptualization, Investigation, Validation.

## Declaration of competing interest

All authors declare that they have no conflicts of interest.

## Data Availability

Data will be made available on request.

## Appendix A Supplementary data

Supplementary file 1. Analytical validation of Baylisascaris procyonis qPCR Image 1

Supplementary file 2. Primers and probes used in three multiplex qPCR assays for canine soil-transmitted helminth detection Image 2
