# Supplementary material for: Surveillance of soil-transmitted helminths and other intestinal parasites in shelter dogs, Mississippi, USA
Source: One Health. 2024 Dec 18;20:100956. doi: 10.1016/j.onehlt.2024.100956 (PMC11743313; doi:10.1016/j.onehlt.2024.100956)
Supplement: Supplementary file 1 — Analytical validation of Baylisascaris procyonis qPCR [file mmc1.docx]

**Supplementary file 1: Analytical Validation of *B. procyonis* qPCR**

Analytical validation of the *Toxocara-B. procyonis* triplex qPCR assay was undertaken using DNA extracts of two *B. procyonis* adults from *Procyon lotor*, and one *Baylisascaris transfugia* adult from *Ursus americanus*. DNA extracts of faecal samples containing *T. canis*, *Spirometra* sp.*, A. caninum, Uncinaria stenocephala, Trichuris vulpis, Dipylidium caninum, Alaria* sp.*, Macracanthorhyncus* sp*., Physaloptera* sp.*, Cystoisosopora* sp.*, Echinococcus granulosis* and *Giardia duodenalis* from domestic dogs (*Canis lupus familiaris*) and a *T. cati* from a domestic cat (*Felis catus*) were used for analytical specificity testing. Ten-fold serial dilutions of the *B. procyonis* female adult DNA extract [at DNA concentration 3.2 ng/μL, determined by Nanodrop (Thermo-Fisher Scientific, Waltham, MA)] and DNA extracted from *T. canis*-infected dingo (*Canis lupus dingo*) faeces at egg count 256 eggs per gram of faeces (epg), and *T. cati*-infected domestic cat faeces at egg count 1120 epg, were used to determine the limit of detection (LoD).

The triplex qPCR amplified all *T. canis*, *T. cati* and *B. procyonis* positive controls. The limit of detection for *Toxocara* spp. from faeces was 2 epg for *T. canis*, 1 epg for *T. cati*. The *Toxocara* targets did not yield amplicons for any of the other parasite DNA extracts tested. No infected raccoon or dog faeces was available for analytical sensitivity testing of the *B. procyonis* target and so DNA extracted from an adult worm was used as a proxy. The limit of detection was found to be 320 pg/mL. The *B. procyonis* target did not yield amplicons for any of the other parasites tested, including *B. transfugia.*
