# Supplementary material for: Surveillance of soil-transmitted helminths and other intestinal parasites in shelter dogs, Mississippi, USA
Source: One Health. 2024 Dec 18;20:100956. doi: 10.1016/j.onehlt.2024.100956 (PMC11743313; doi:10.1016/j.onehlt.2024.100956)
Supplement: Supplementary file 2 — Primers and probes used in three multiplex qPCR assays for canine soil-transmitted helminth detection [file mmc2.docx]

**Supplementary file 2**. Primers and probes used in three multiplex qPCR assays for canine soil-transmitted helminth detection.

| **Multiplex qPCR** | **Target** | **Primer/Probe** | **Sequence (5’-3’)** | **Size (bp)** | **Target gene** | **Final conc (nM)** | **References** |
| --- | --- | --- | --- | --- | --- | --- | --- |
| qPCR #1 | Canine DNA | MAM-F | CGA CCT CGA TGT TGG ATC AG | 92 | 16S Mt rRNA | 50 | [28] |
|  |  | MAM-R | GAA CTC AGA TCA CGT AGG ACT TT |  |  | 50 |  |
|  |  | MAM PROBE | 5Cy5/CCT AAT GGT/ TAO/ GCA GCA GCT ATT AA/ 3IAbRQSp |  |  | 100 |  |
|  | Equine Herpesvirus – 4 | EHV-FWD | GAT GAC ACT AGC GAC TTC GA | 81 | Glycoprotein B | 40 |  |
|  |  | EHV-REV | TTT CGC GTG CCT CCT CCA G |  |  | 40 |  |
|  |  | EHV PROBE | ROX/TTT CGC GTG CCT CCT CCA G/3IAbRQSp |  |  | 100 |  |
|  | *A. caninum* & *A. ceylanicum* | Acancey-F | GGG AAG GTT GGG AGT ATC G | 103 | ITS-1 rRNA | 300 |  |
|  |  | Acancey-R | CGA ACT TCG CAC AGC AAT C |  |  | 300 |  |
|  | *A. caninum* | Acan probe | 5HEX/AG+T+CGT+T+A+C+TGG/3IABkFQ |  |  | 100 |  |
|  | *A. ceylanicum* | AceyDOGprobe | 56-FAM/CCGTTC+CTGGGTGGC/3IAbRQSp |  |  | 100 |  |
| qPCR #2 | Equine Herpesvirus – 4 | EHV-FWD | GAT GAC ACT AGC GAC TTC GA | 81 | Glycoprotein B | 40 |  |
|  |  | EHV-REV | TTT CGC GTG CCT CCT CCA G |  |  | 40 |  |
|  |  | EHV PROBE | ROX/TTT CGC GTG CCT CCT CCA G/3IAbRQSp |  |  | 100 |  |
|  | *A.braziliense* & *U. stenocephala* | Uncbraz-F | GAG CTT TAG ACT TGA TGA GCA TTG |  | ITS-1 rRNA | 350 |  |
|  |  | Uncbraz-R | GCA GAT CAT TAA GGT TTC CTG AC |  |  | 350 |  |
|  | *A. braziliense* | AbraProbe | 56FAM/TGA GCG CTA /ZEN/GGC TAA CGC CT/3IABkFQ | 119 |  | 100 |  |
|  | *U. stenocephala* | UncProbe | 5HEX/CAT TAG GCG /ZEN/GCA ACG TCT GGT G/3IABkFQ | 118 |  | 100 |  |
|  | *Strongyloides* | StrF | CCA AGT AAA CGT AAG TCA TTA GC | 101 | 18S rRNA | 50 | [29] |
|  |  | StrR | CGC CTC TGG ATA TTG CTC AGT TCC |  |  | 50 |  |
|  |  | STR Probe | 5Cy5/ACA CAC CGG/TAO/CCG TCG CTG C/3IAbRQSp |  |  | 150 |  |
| qPCR #3 | *T. canis* | Tcan-F | GCG CCA ATT TAT GGA ATG TGA T | 141 | ITS-2 rRNA | 300 | [30] |
|  |  | Tox-R | GAG CAA ACG ACA GCS ATT TCT T |  |  | 300 |  |
|  |  | Tcan-Pr | 56-FAM/CC ATT ACC A/ZEN/C ACC AGC ATA GCT CAC CGA /3IABkFQ |  |  | 100 |  |
|  | *T. cati* | Tcat-F | ACG CGT ACG TAT GGA ATG TGC T | 155 |  | 300 |  |
|  |  | Tox-R | GAG CAA ACG ACA GCS ATT TCT T |  |  | 300 |  |
|  |  | Tcat-Pr* | 56-JOE/TC TTT CGC A/ZEN/A CGT GCA TTC GGT GA/3IABkFQ |  |  | 100 |  |
|  | *B. procyonis* | Bpro-F | GAG TTA TGA GTT TAG TGA TAT TCC TGG A | 157 | *cox2* | 250 | [31] |
|  |  | Bpro-R | GCA AAG CCC AAG AAT GAA TCA C |  |  | 250 |  |
|  |  | Bpro-Pr* | 56-ROX/TA TTA ACA TCA CAA GGT ACA ACA CAA CG/3IAbRQSp |  |  | 100 |  |
| +, Locked nucleic acid (LNA) bases; *, Modifications made to the published probe | | | | | | | |
